# Supplementary material for: Gene identification and RNAi-silencing of p62/SQSTM1 in the vector Rhodnius prolixus reveals a high degree of sequence conservation but no apparent deficiency-related phenotypes in vitellogenic females
Source: PLoS One. 2023 Jul 24;18(7):e0287488. doi: 10.1371/journal.pone.0287488 (PMC10365311; doi:10.1371/journal.pone.0287488)
Supplement: S1 Table — All sequences were obtained from Vector Base (https://www.vectorbase.org/) and primers were synthesized by Macrogen or IDT technologies. (DOCX) [file pone.0287488.s001.docx]

**Table S1: Genes and primers List.** All sequences were obtained from *Vector Base* (<https://www.vectorbase.org/>) and primers were synthesized by Macrogen or IDT technologies. T7 adaptor sequences are underlined.

| **GENE** | **VECTOR**  **BASE** | **PRIMER SEQUENCE (5’-3’)** | **AMPLICON**  **(bp)** | **PRIMER EFFICIENCY** |
| --- | --- | --- | --- | --- |
| **18S** | RPRC017412 | FOR: TCGGCCAACAAAAGTACACA  REV: TGTCGGTGTAACTGGCATGT | 105 | [1] |
| **p62 (qPCR)** | New annotated gene in this study | FOR: AATGACGTTTTGAAGGCGGG  REV: TTGCTCGATGTTGCCGTTTT | 225 | **Slope:** -3,486  **Y-inter:** 26,924  **R2:** 0,982  **Efficiency %:** 93.592 |
| **p62 (dsRNA)** |  | FOR: TAATACGACTCACTATAGGGTACTCCGTCCACATCAACCAACAG  REV: TAATACGACTCACTATAGGGTACTGGTGAAGTTGATGGAGTGGC | 606 | - |
| **T7 adaptor** | - | FOR: GAGAATTCTAATACGACTCACTATAGGGCCGCGG  REV: AGGGATCCTAATACGACTCACTATAGGGCCCGGGGC | - | - |

1. Majerowicz D, Alves-Bezerra M, Logullo R, Fonseca-De-Souza AL, Meyer-Fernandes JR, Braz GRC, et al. Looking for reference genes for real-time quantitative PCR experiments in Rhodnius prolixus (Hemiptera: Reduviidae). Insect Mol Biol. 2011;20: 713–722. doi:10.1111/j.1365-2583.2011.01101.x
