# Supplementary figures and images for: Gene identification and RNAi-silencing of p62/SQSTM1 in the vector Rhodnius prolixus reveals a high degree of sequence conservation but no apparent deficiency-related phenotypes in vitellogenic females
Source: PLoS One. 2023 Jul 24;18(7):e0287488. doi: 10.1371/journal.pone.0287488 (PMC10365311; doi:10.1371/journal.pone.0287488)

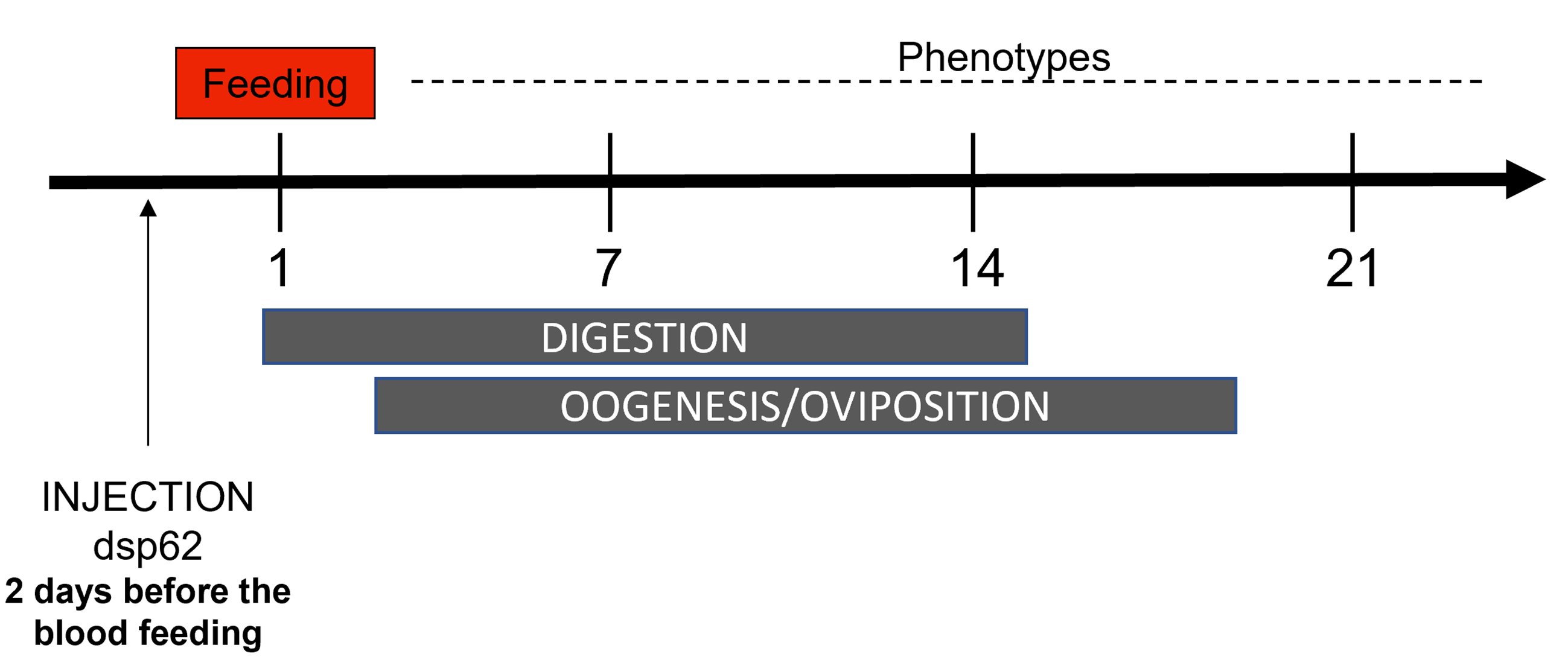

Supplement: S1 Fig — Mated females of the second or third blood feeding after emerging as adults were injected with dsRNAs 2 days before their blood feeding and the generated phenotypes were observed over the following gonotrophic cycle. (TIF) [file pone.0287488.s002.tif]

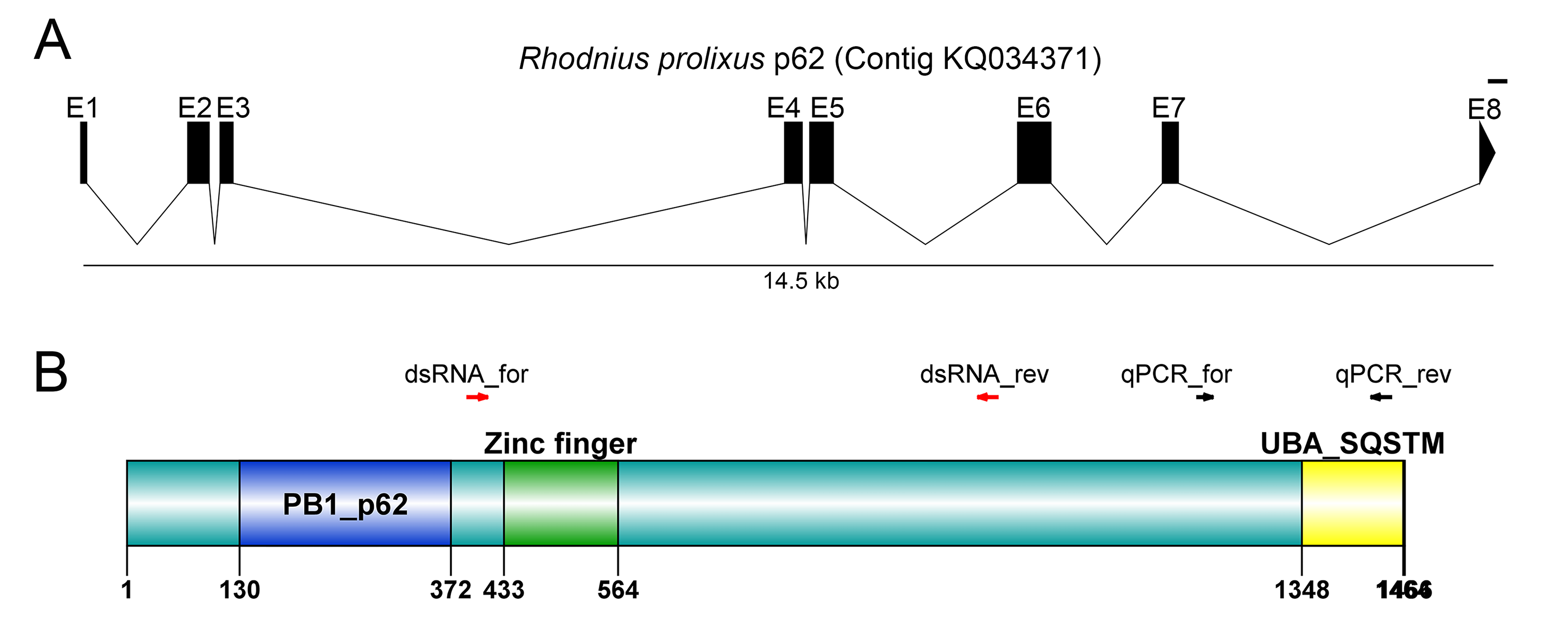

Supplement: S2 Fig — Schematic diagram showing exons and introns were generated using the exon-intron graphic maker available at wormweb.org/exonintron. Sequence information was obtained from the raw genome data at Vector Base (https://www.vectorbase.org/). B. Schematic diagram showing conserved domains and primers targeting regions. The graphic was generated using the DOG 2.0 software (https://dog.biocuckoo.org). Conserved domains were identified using Pfam 35.0 (https://pfam.xfam.org/). C. Predicted model of R. prolixus p62 protein using the software ITASSER. (TIF) [file pone.0287488.s003.tif]

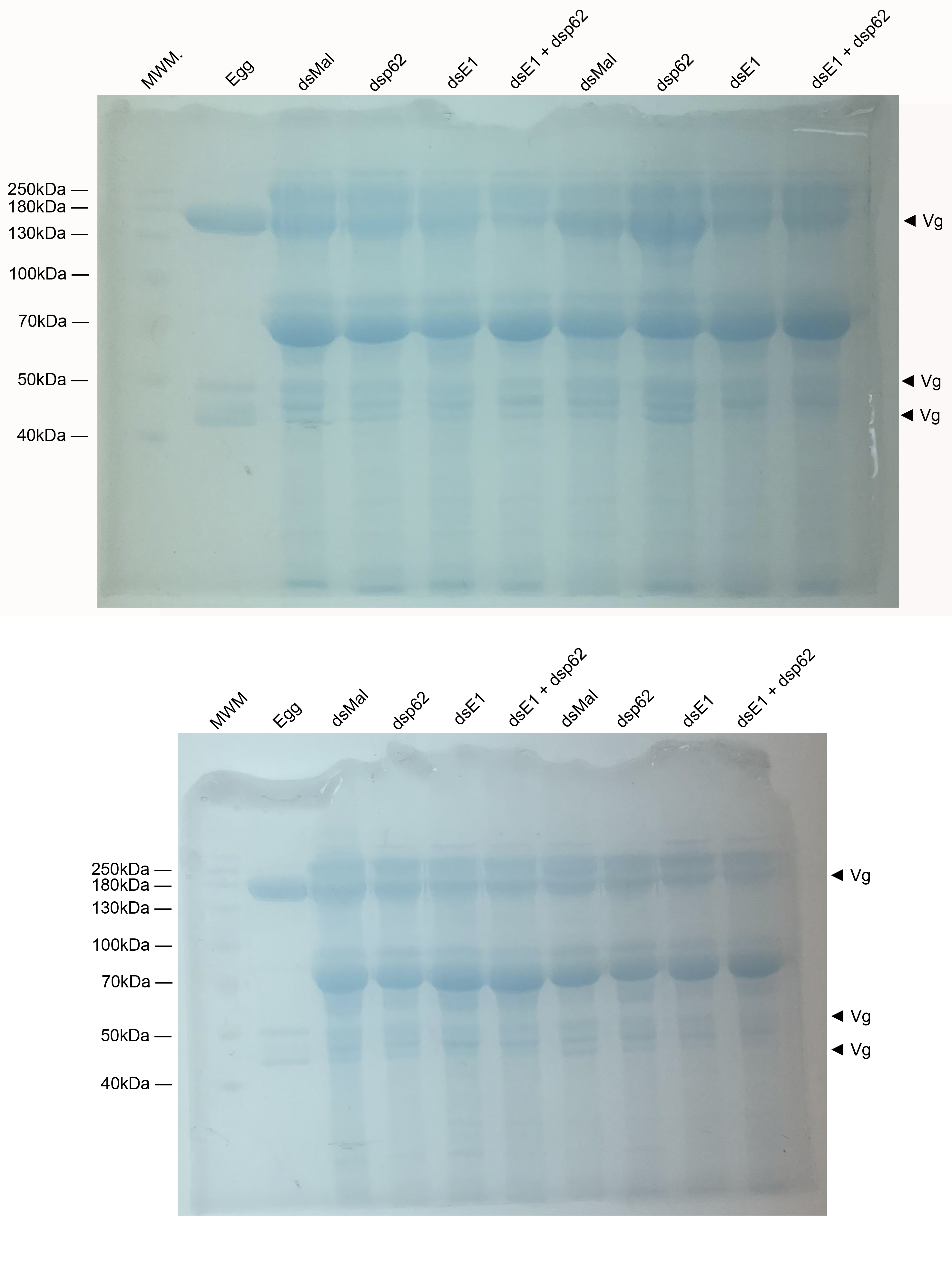

Supplement: S3 Fig — Hemolymph protein profiles shown in a 10% SDS-PAGE for control and silenced females. Arrowheads point to the main yolk protein vitellogenin (Vg) (n = 4). MWM, molecular weight markers in kDa. Egg, 30°g of protein extracted from eggs laid 24h after oviposition. Arrowheads point to the vitellogenin subunits. (TIF) [file pone.0287488.s004.tif]

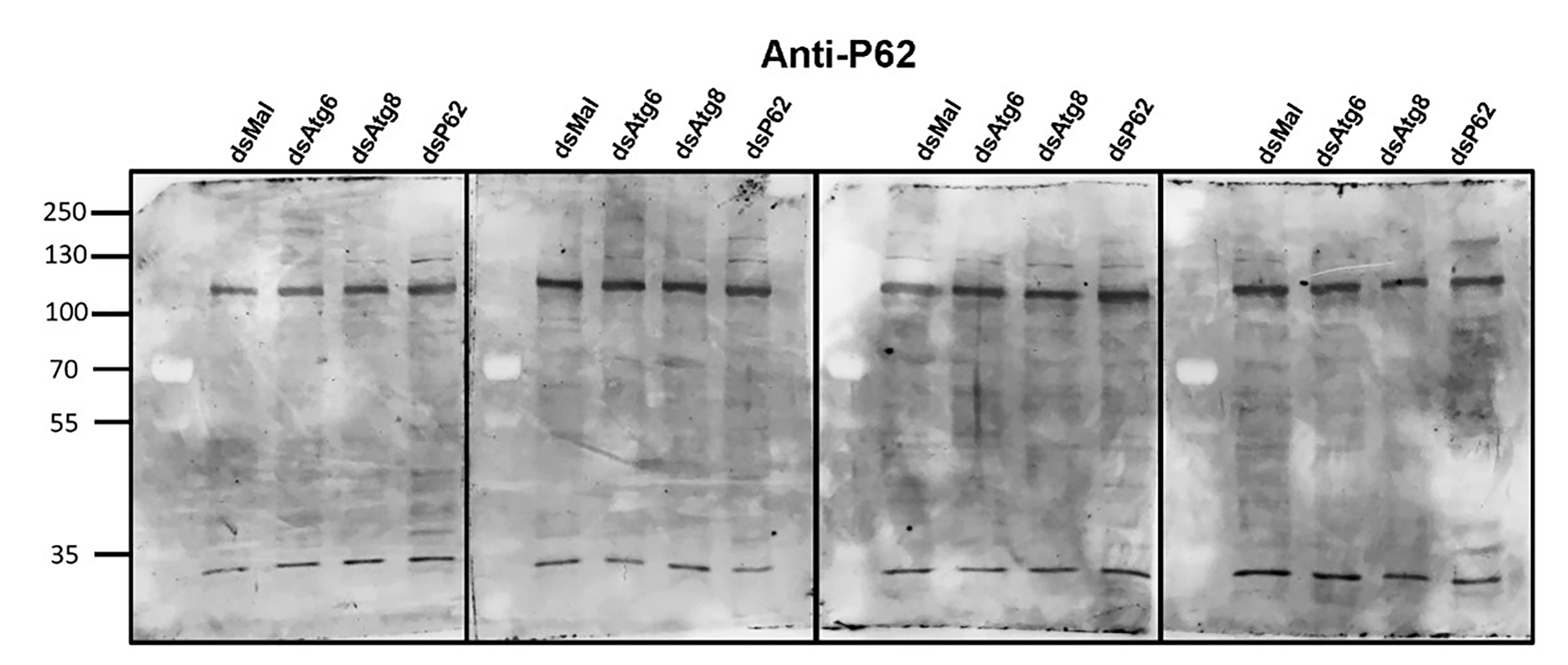

Supplement: S4 Fig — A 14-amino acid peptide of R. prolixus p62 was synthesized and injected in rats. The final bleed was used for immunoblottings against ATG6- and ATG8-silenced samples, as well as p62-silenced samples. Each panel represents a different experiment (N = 4). The numbers on the left indicate the molecular weight marker indications in kDa. (TIF) [file pone.0287488.s005.tif]
