# Supplementary material for: Gene identification and RNAi-silencing of p62/SQSTM1 in the vector Rhodnius prolixus reveals a high degree of sequence conservation but no apparent deficiency-related phenotypes in vitellogenic females
Source: PLoS One. 2023 Jul 24;18(7):e0287488. doi: 10.1371/journal.pone.0287488 (PMC10365311; doi:10.1371/journal.pone.0287488)
Supplement: Attachment — Submitted filename: p62_authors answers_R1.pdf [file pone.0287488.s006.pdf]

R1- Manuscript #PONE-D-22-35091

Journal Requirements:

When submitting your revision, we need you to address these additional requirements.
1. Please ensure that your manuscript meets PLOS ONE's style requirements, including those for file naming. The PLOS ONE style templates can be found at https://journals.plos.org/plosone/s/file?id=wjVg/PLOSOne_formatting_sample_main_body.pdf and https://journals.plos.org/plosone/s/file?id=ba62/PLOSOne_formatting_sample_title_authors_affiliations.pdf
Author´s answer: Done.

2. We noticed you have some minor occurrence of overlapping text with the following previous publication(s), which needs to be addressed:
https://onlinelibrary.wiley.com/doi/10.1002/jcp.30806
In your revision ensure you cite all your sources (including your own works), and quote or rephrase any duplicated text outside the methods section. Further consideration is dependent on these concerns being addressed.
Author´s answer: The minor overlaps were rephrased.

3. We note that the grant information you provided in the 'Funding Information' and 'Financial Disclosure' sections do not match.
When you resubmit, please ensure that you provide the correct grant numbers for the awards you received for your study in the 'Funding Information' section.
Author´s answer: Done.

4. Please expand the acronym "JCNE-FAPERJ, INCT-EM-CNPq/FAPERJ, CAPES" (as indicated in your financial disclosure) so that it states the name of your funders in full.
This information should be included in your cover letter; we will change the online submission form on your behalf.
Author´s answer: Done. Information added to the cover letter.

5. Thank you for stating the following in the Acknowledgments Section of your manuscript:
"The authors thank CENABIO-UFRJ and the funding agencies FAPERJ, CNPq and CAPES"
We note that you have provided funding information that is not currently declared in your Funding Statement. However, funding information should not appear in the Acknowledgments section or other areas of your manuscript. We will only publish funding information present in the Funding Statement section of the online submission form.
Please remove any funding-related text from the manuscript and let us know how you would like to update your Funding Statement. Currently, your Funding Statement reads as follows:
"This research was funded by the following grants: JCNE-FAPERJ (www.faperj.br/), INCT-EM-CNPq/FAPERJ (http://cnpq.br/) and CAPES (www.capes.gov.br/) to I.R. The funders had no role in study design, data collection and analysis, decision to publish, or preparation of the manuscript."

Please include your amended statements within your cover letter; we will change the online submission form on your behalf.
Author´s answer: The funding information was deleted from the acknowledgements.
The funding statement and financial disclosure are now corrected and added to the cover letter: "This research was funded by the following grants: Jovem Cientista do Nosso Estado (JCNE)-Fundação de Amparo à Pesquisa do Estado do Rio de Janeiro (FAPERJ) (www.faperj.br/),

Instituto Nacional de Ciência e Tecnologia em Entomologia Molecular (INCT-EM) - Conselho Nacional de Desenvolvimento Científico e Tecnológico (CNPq) (http://cnpq.br/) and Coordenação de Aperfeiçoamento de Pessoal de Nível Superior (CAPES) (www.capes.gov.br/) to I.R. The funders had no role in study design, data collection and analysis, decision to publish, or preparation of the manuscript."

6. PLOS ONE now requires that authors provide the original uncropped and unadjusted images underlying all blot or gel results reported in a submission's figures or Supporting Information files. This policy and the journal's other requirements for blot/gel reporting and figure preparation are described in detail at https://journals.plos.org/plosone/s/figures#loc-blot-and-gel-reporting-requirements and https://journals.plos.org/plosone/s/figures#loc-preparing-figures-from-image-files. When you submit your revised manuscript, please ensure that your figures adhere fully to these guidelines and provide the original underlying images for all blot or gel data reported in your submission. See the following link for instructions on providing the original image data: https://journals.plos.org/plosone/s/figures#loc-original-images-for-blots-and-gels.
In your cover letter, please note whether your blot/gel image data are in Supporting Information or posted at a public data repository, provide the repository URL if relevant, and provide specific details as to which raw blot/gel images, if any, are not available. Email us at plosone@plos.org if you have any questions.
Author´s answer: The original uncropped and unadjusted images of the SDS-PAGEs were added to the supporting information in the revised manuscript (Fig S3). This was stated in the cover letter.

7. Please upload a new copy of Figure 1 and 8 as the detail is not clear. Please follow the link for more information:
https://blogs.plos.org/plos/2019/06/looking-good-tips-for-creating-your-plos-figures-graphics/
https://blogs.plos.org/plos/2019/06/looking-good-tips-for-creating-your-plos-figures-graphics/
Author´s answer: The new copies of fig 1 and Fig 8 were uploaded.

8. Please include captions for your Supporting Information files at the end of your manuscript, and update any in-text citations to match accordingly. Please see our Supporting Information guidelines for more information: http://journals.plos.org/plosone/s/supporting-information.
Author´s answer: Done.

Additional Editor Comments:

The reviewers have indicated that there are considerable issues that must be addressed before reconsideration. This will likely require a combination of extensive editing and some additional analyses. Importantly, the reviewers had a split decision on acceptance (one suggested revision and the other suggested reject), so the manuscript will be sent for review again after revision.
Author´s answer to the editor: The manuscript formatting was corrected, and all issues pointed by the reviewers were addressed below.

Reviewer #1: General comments:

The manuscript by Pereira et al. studies the autophagy/UPS adaptor protein p62 in the hematophagous model insect Rhodnius prolixus, a vector of Chagas disease. To do so, the authors employed biochemical and molecular biological approaches, including gene silencing. The article addresses an interesting topic, as little information is available on p62 in insects, apart from what has been reported in Drosophila. The manuscript is well organized and written. The objectives and experimental design are clear and straightforward.

Author´s answer: We thank the reviewer for the careful revision and for the positive appreciation.

Broadly, the article is divided into two parts, the first descriptive and the second functional. Although the gene description (sequence, domain, phylogenetic analysis) is well done, I am concerned that the manuscript fails to attribute a functional role to p62 as the evidence obtained is negative. The authors should have performed additional experiments explaining why silencing the p62 gene did not produce any observable phenotype.
Author´s answer: We have added new experiments and discussion regarding the absence of apparent phenotypes. Please see the information below.

Specific comments:
Pages 11-12, section "Parental RNAi silencing of p62...": The authors mention three possible explanations for why the silenced insects showed no change in phenotype, but did not pursue those options. A quick Internet search shows several major antibody suppliers providing anti-p62 polyclonal options that could cross-react against the Rhodnius protein. There is even an antibody that is described as reacting against "invertebrate" p62 and is therefore worth testing (https://www.novusbio.com/products/p62-sqstm1-antibody_nbp1-48320).
Author´s answer: Agreed. Regarding the antibodies, we failed to add to the original manuscript the data that we already had with custom-made antibodies raised against *R. prolixus* p62. Please see below. Regarding the other hypotheses, we did try to pursue them with the available tools for this model. They are now better discussed in the revised manuscript. All is explained below.

Explanation 1) <u>We cannot rule out the possibility that indeed there is no apparent deficiency phenotypes arising from the silencing of this gene under our conditions of insect-rearing and observation.</u> This possibility was better discussed in the revised manuscript. The new text added to the manuscript is copied below:
"Regarding hypothesis number one, one should consider the possibility that the function of p62 might be necessary under conditions different from those in which we normally keep our insects. Changes in environmental conditions (temperature, humidity, photoperiod, etc.), nutritional status (different cycles and types of diet), immunological stress (exposure to pathogens, symbionts, etc.), among many other factors, can alter the general physiology of individuals at the molecular level, exposing the specific function of a certain gene (Schmidt-Nielsen, 1997; Hoffmann, 2012; Zhang et al., 2019). In this case, we cannot rule out the hypothesis that p62 function can emerge as required under a certain condition that is not met under our in-house conditions."

Explanation 2) <u>mRNA silencing was efficient (which we tested), but protein decrease was not.</u> To address this possibility, we agree that accessing the protein levels using antibodies would be vital for improved interpretations of our findings, and we did test antibodies raised against a synthetic peptide of *R. prolixus* p62. The data and its methodology was included in the revised manuscript as Fig S4. The text (included in the results and discussion section) is copied below:
"To address the second hypothesis, accessing the p62 protein levels using reliable antibodies would be vital for improved interpretations of our findings. Still, our experimental options are limited to the methods available for a non-model organism such as *Rhodnius prolixus*, and, in our hands, commercial cross-species antibodies usually do not generate consistent results. For this reason, we ordered custom made antibodies against *R. prolixus* p62 raised against a synthetic N-terminal peptide of 14 AAs of the R. prolixus p62 protein. The final bleed, with an ELISA title of 1:512.000, was used for immunoblottings with our samples in ATG6- (Vieira et al., 2018b) and ATG8-silenced (Pereira et al., 2020) insects, as well as p62-silenced samples. Because p62 is typically used as read out of autophagic flux, it is expected that the silencing of ATGs would result in an increase in the p62 fragment (indicating decreased autophagic flux) (Klionsky

et al., 2021). Unfortunately, the raised antiserum labeled two bands, and none of them matched the expected *R. prolixus* p62 molecular weight of approximately 50 kDa. Furthermore, none of the gene-silencings described above resulted in alterations in the detected bands, pointing to unspecific labeling. Thus, unfortunately, we could not follow through with the antibodies and direct testing of this hypothesis."

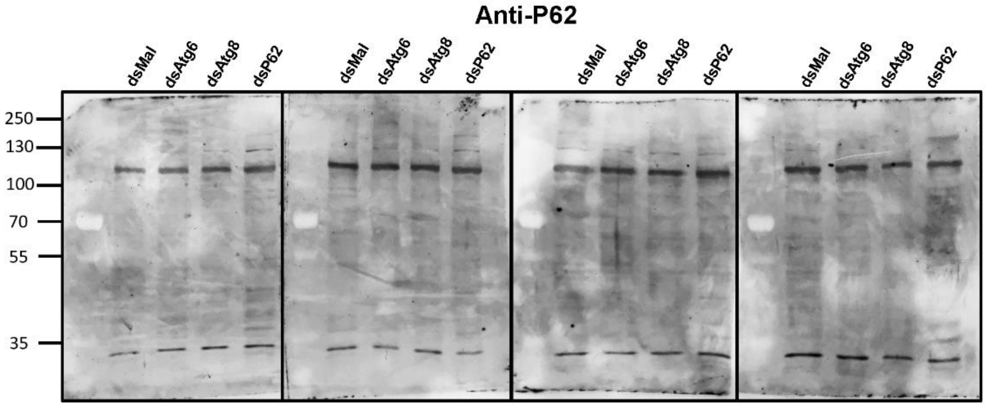

Fig S4. Immunoblotting against *R. prolixus* p62. Four independent experiments.

In addition, we have performed new qPCRs to test if the silencing of p62 mRNA was persistent until the end of the analyzed gonotrophic cycle (21 days after the blood meal). We found that the p62 mRNA was still silenced after 14 and 21 days after the blood meal. Thus, the hypothesis of residual protein levels impairing the observation of phenotypes would arise from unusual high levels of stability of the p62 protein, and not from a transient mRNA silencing. This data (qPCR - p62 mRNA silencing at days 14 and 21 after the blood meal) was added to the manuscript as Fig 1D and discussed.
The revised text including the discussion on the new qPCRs is copied below:
"Nevertheless, even while we can't completely rule out the hypothesis that the p62 protein was not downregulated, it is crucial to note that the persistent mRNA silencing (until 21 days after the blood feeding, 23 days after the dsRNA injection, Fig 1D) would require unusual high levels of protein stability to sustain function for over the 21 days of gonotrophic cycle."

Explanation 3) <u>Silencing of p62 triggered compensatory mechanisms that allowed the insect to adapt and accomplish general physiology tasks as efficiently as control individuals.</u>
This hypothesis was pursued and discussed by testing the modulation of autophagy and UPR markers.

Since the title of the article reports that this is a "functional characterization" of the p62, more experiments that attempt to shed light on the function of the protein should be included.
Author´s answer: The supplementary experiments to attempt to shed light on p62´s function were described above. However, we agree that the term functional characterization in the title does not match the findings. So, we changed the title to: "Gene identification and RNAi-silencing of p62/SQSTM1 in the vector *Rhodnius prolixus* reveals a high degree of sequence conservation but no apparent deficiency-related phenotypes in vitellogenic females".

- Page 8, first paragraph: citations are missing. Please include them.
Author´s answer: Done. The following references were added.
(Nunes-da-fonseca et al., 2017; World Health Organization, 2019; Eberhard et al., 2020; Lange et al., 2022)

- Page 10, first line: "melpomene" should not be in capital letters. Please correct.
Author´s answer: Done**.**

- Page 10, second line: "lectularius" is misspelled. Please correct.
Author´s answer: Done**.**

- Page 10, third line: "Daphnia" is misspelled. Please correct.
Author´s answer: Done**.**

- Table S1: The word "list" should not be capitalized. Please correct.
Author´s answer: Done.

- Page 10, RNAi silencing section: There is a space missing between "606" and "bp". Please add. Please revise this aspect (space between magnitudes and their respective units) throughout the manuscript.
Author´s answer: Done and corrected throughout the revised manuscript.

- Page 11, "Yolk organelles..." section: No need to capitalize "flow cytometry" in the title.
Author´s answer: Corrected.

- Fig. 1 has low resolution. Please upload an improved image.
Author´s answer: Done. We apologize for the poor resolution image.

- Page 11, "The single isoform..." section, last paragraph: "R. prolixus" needs to be italicized. Please correct.
Author´s answer: Corrected.

Reviewer #2: The authors propose the genomic and transcriptional characterization of the protein p62 and the study of its relevance during the vitellogenesis of the Chagas 'disease vector Rhodnius prolixus. In my opinion, the manuscript only resolves the aspect related with the genomic characterization of the protein.
Author´s answer: We thank the reviewer for the comments. All points raised were addressed below. The authors would like to note, however, that this manuscript describes the identification of a new gene, its transcription profile in the different organs and during oogenesis of the vector of an important neglected tropical disease, as well as the data resultant from its RNAi silencing. Although we did not observe apparent phenotypes resultant from the gene silencing to allow conclusions regarding this gene functional role, the manuscript presents original research performed to a high technical standard. Thus, respectfully, we consider the data worth of publishing, and we chose the journal Plos One due to its "mission to publish all valid research, considering negative and null results" Please see https://journals.plos.org/plosone/s/criteria-for-publication.
Thus, we appreciate the reviewer´s time to evaluate again the manuscript considering the explanations and corrections in the revised manuscript.

Moreover, the phylogenetic analysis is not representative. The authors claim that the identity of the protein is confirmed based on its occurrence in same clade with Drosophila melanogaster but, at the same time is true that Daphnia pulex (Crustacea) appears in the clade more proximal to the hemipteran species than D. melanogaster and Anopheles gambiae.
Author´s answer: We define each clade based on the branches originating at the tree's base, identified with different colors. The high bootstrap values in the blue, red, and green clades provide statistical support for the hypothesis that the proteins in each of these clades are of

different origins. On the other hand, for example, the *D. pulex* DAPPUDRAFT 339667 protein was external to the clades and could not be identified based on other genomes. Therefore, we used the *D. melanogaster* genome annotations as a guide, as this genome has the most reliable annotation. Based on this, we identified all red clade proteins as p62 orthologs in their respective organisms. The same goes for the other clades. However, statistical support is low within each clade, which does not allow us to state with certainty the relationships between these proteins.

Furthermore, it is not clear the reasons why the authors include in the phylogenetic analysis other related proteins. It seems to be not necessary. Otherwise, each one of these analysis involving other proteins, if are trully relevant, should be performed independently of the P62 analysis. Finally, the trees in the current form should not be included.
Author´s answer: We included other proteins in the analysis to perform an unbiased approach. Our approach included all PB1 domain proteins, including p62 and others, present in the analyzed genomes. This approach obliges us to analyze all the proteins, preventing the researcher from introducing bias if he searches with non-automated criteria. For example, we could have searched for Blast using a p62 protein as a query and included the hits obtained in the analysis. However, depending on the chosen inclusion criteria, many other unrelated proteins could have been included (if the inclusion criteria were not strict), or we could not discover the duplications seen in *A. gambiae* and *D. pulex* (if the inclusion criteria inclusion was too strict). Our approach avoids these types of problems.

Furthermore, the authors claim that the gene is absent in Aedes aegypti and other species pertaining to other insect orders. It seems not probable the the protein could be lost in Ae. aegypti, being present in the related genus Anopheles. Indeed, it seems not probable that this protein could be lost along insect evolution. It would more probable that the authors didn´t find the corresponding sequences in the genomes.
Author´s answer: It is possible that the genomes of some insects do not have the missing proteins annotated, and, by our search strategy, we would not have found them. However, nothing prevents the gene from being lost in *A. aedes*, and not in *A. gambiae*. Gene loss has happened several times during insect diversification (Eirín-López et al., 2012). These hypotheses are mentioned in the revised manuscript:
"On the other hand, the gene is apparently absent in *A. aegypti*, *H. melpomene*, *T. castaneum*, and *A. pisum*. Whether the gene was lost in the evolutionary process or was just not annotated in the genomes needs to be further investigated (Fig 1, red clade)."

Regarding the experimental designs, a number of situations are not clear. Why the authors used females after two or three feeding cycles. Why didn´t use females on the first cycle? I assume that on the first cycle they can get a more accurate standardization of the individuals used for experiments.
Author´s answer: All females used in this work were obtained from our insectarium where mated females are fed for the first time (as adult insects) in live-rabbit blood 14 days after the 5th instar nymph to adult ecdysis. During the first blood feeding cycle, the insects are kept back generating the eggs that will hatch as first instar nymphs to maintain the insectarium. After the first blood feeding, all adult insects are fed every 21 days and only fully gorged insects are used for the experiments. Thus, females of the second or third blood feeding were used, and they are highly synchronized regarding blood feeding, digestion, and oviposition. Functional, molecular and physiology studies have been performed using these insects over the past decades. A few examples follow (Masuda and Oliveira, 1985; Oliveira et al., 1989, 2000, 2017, 2018; Moreira et al., 2003; Bouts et al., 2007; Walter-Nuno et al., 2013; Sterkel et al., 2016; Brito et al., 2018; Vieira et al., 2018a, 2021; Pereira et al., 2022, 2020b; Rios et al., 2021; Silva-Oliveira et al., 2021; Entringer et al., 2021)

It is also not clear, when the silencing treatments were done. RNAi silencing treatment of unfed females means first blood feeding as adults as it can be interpreted from M&M section? It means that experimental data were taken after more than 30 days after treatment? Any way, if the females were fed three times, is possible to analyse them in a synchronized physiological condition?

Author´s answer: We apologize for not making it clearer. The insects were injected 2 days before their blood feeding (for the second or third time that they were feeding as adults), and the phenotypes were observed over the following gonotrophic cycle. The only data acquired for more than 30 days after the dsRNA injection (28 days after blood feeding) was the survival data. To make it clearer, we have added the timeline of our experimental design as Fig S1.

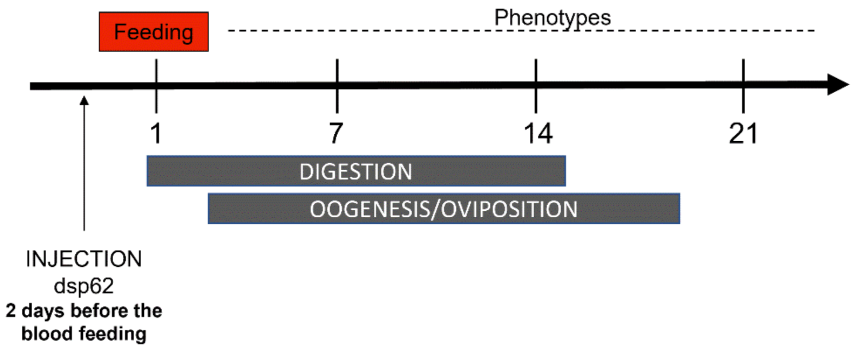

Also, we have added the data of p62 gene silencing at 14 and 21 days after feeding, and found that its silencing efficiency remained unaltered over that period (please see Fig 2D in the revised manuscript). Thus, all phenotypes were analyzed under conditions where p62 mRNA was silenced.

The authors also claim that p62 might be not relevant under in-house conditions! Is this possible? Are the physiology of the insects different in house-conditions?

Author´s answer: We meant that one should consider the possibility that the function of p62 might be necessary under conditions different from those in which we normally keep our insects. Changes in environmental stress conditions (temperature, humidity, photoperiod, etc.), nutritional stress (different cycles and types of diet, in the case of *R. prolixus*, blood from different hosts, for example), immunological stress (exposure to pathogens, symbionts, etc.) among many other factors, can alter the physiology of individuals exposing the specific function of a certain gene. We have made this point clearer in the discussion of the revised manuscript. The new text is copied below:

"Regarding hypothesis number one, one should consider the possibility that the function of p62 might be necessary under conditions different from those in which we normally keep our insects. Changes in environmental conditions (temperature, humidity, photoperiod, etc.), nutritional status (different cycles and types of diet), immunological stress (exposure to pathogens, symbionts, etc.), among many other factors, can alter the general physiology of individuals at the molecular level, exposing the specific function of a certain gene (Schmidt-Nielsen, 1997; Hoffmann, 2012; Zhang et al., 2019). In this case, we cannot rule out the hypothesis that p62 function can emerge as required under a certain condition that is not met under our in-house conditions"

comments:

- "Heliconius Melpomene" must be "Heliconius melpomene"

Author´s answer: Corrected.

- "Cimex letularius" is "Cimex lectularius"

Author´s answer: Corrected.

- "Daphia pulex" should be changed by "Daphnia pulex"
Author´s answer: Corrected.

Finally, we found that the manuscript present both experimental and theoretical failures to be published in the current form.
Author´s answer: The alleged experimental and theoretical failures were clarified above.

References:

Bouts, D. M. D., do Amaral Melo, A. C., Andrade, A. L. H., Silva-Neto, M. A. C., de Oliveira Paiva-Silva, G., Sorgine, M. H. F., et al. (2007). Biochemical properties of the major proteins from Rhodnius prolixus eggshell. *Insect Biochem. Mol. Biol.* 37, 1207–1221.

Brito, T., Julio, A., Berni, M., de Castro Poncio, L., Bernardes, E. S., Araujo, H., et al. (2018). Transcriptomic and functional analyses of the piRNA pathway in the Chagas disease vector Rhodnius prolixus. *PLoS Negl. Trop. Dis.* 12, e0006760.

Eberhard, F. E., Cunze, S., Kochmann, J., and Klimpel, S. (2020). Modelling the climatic suitability of Chagas disease vectors on a global scale. *Elife* 9, e52072. doi:10.7554/eLife.52072.

Eirín-López, J. M., Rebordinos, L., Rooney, A. P., and Rozas, J. (2012). The birth-and-death evolution of multigene families revisited. *Repetitive DNA* 7, 170–196.

Entringer, P. F., Majerowicz, D., and Gondim, K. C. (2021). The fate of dietary cholesterol in the kissing bug Rhodnius prolixus. *Front. Physiol.* 12, 654565.

Hoffmann, K. H. (2012). *Environmental physiology and biochemistry of insects*. Springer Science & Business Media.

Klionsky, D. J., Abdel-Aziz, A. K., Abdelfatah, S., Abdellatif, M., Abdoli, A., Abel, S., et al. (2021). Guidelines for the use and interpretation of assays for monitoring autophagy. *Autophagy* 17, 1–382.

Lange, A. B., Leyria, J., and Orchard, I. (2022). The hormonal and neural control of egg production in the historically important model insect, Rhodnius prolixus: A review, with new insights in this post-genomic era. *Gen. Comp. Endocrinol.* 321–322. doi:10.1016/j.ygcen.2022.114030.

Masuda, H., and Oliveira, P. L. (1985). Characterization of vitellin and vitellogenin of Rhodnius prolixus. identification of phosphorilated compounds in the molecule . *Insect Bichemistry Mol. Biol.* 15, 543–550.

Moreira, M. F., Coelho, H. S. L., Zingali, R. B., Oliveira, P. L., and Masuda, H. (2003). Changes in salivary nitrophorin profile during the life cycle of the blood-sucking bug Rhodnius prolixus. *Insect Biochem. Mol. Biol.* 33, 23–28.

Nunes-da-fonseca, R., Berni, M., Pane, A., and Araujo, H. M. (2017). Rhodnius prolixus : From classical physiology to modern developmental biology. 1–11. doi:10.1002/dvg.22995.

Oliveira, D. S., Brito, N. F., Franco, T. A., Moreira, M. F., Leal, W. S., and Melo, A. C. A. (2018). Functional characterization of odorant binding protein 27 (RproOBP27) from Rhodnius prolixus antennae. *Front. Physiol.* 9, 1175.

Oliveira, J. H. M., Talyuli, O. A. C., Goncalves, R. L. S., Paiva-Silva, G. O., Sorgine, M. H. F., Alvarenga, P. H., et al. (2017). Catalase protects Aedes aegypti from oxidative stress and increases midgut infection prevalence of Dengue but not Zika. *PLoS Negl. Trop. Dis.* 11, 1–13. doi:10.1371/journal.pntd.0005525.

Oliveira, M. F., Silva, J. R., Dansa-Petretski, M., de Souza, W., Braga, C. M. S., Masuda, H., et al. (2000). Haemozoin formation in the midgut of the blood-sucking insect Rhodnius prolixus. *Febs Lett.* 477, 95–98.

Oliveira, P. L., Petretski, M. D. A., and Masuda, H. (1989). Vitelling processing and degradation

during embryogenesis of Rhodnius prolixus. *Insect Biochem* 19, 489–498.
Pereira, J., Dias, R., and Ramos, I. (2022). Knockdown of E1- and E2-ubiquitin enzymes triggers defective chorion biogenesis and modulation of autophagy-related genes in the follicle cells of the vector Rhodnius prolixus. *J. Cell. Physiol.* 1, 12.
Pereira, J., Diogo, C., Fonseca, A., Bomfim, L., Cardoso, P., Santos, A., et al. (2020). Silencing of RpATG8 impairs the biogenesis of maternal autophagosomes in vitellogenic oocytes, but does not interrupt follicular atresia in the insect vector Rhodnius prolixus. *PLoS Negl. Trop. Dis.* 14, e0008012. doi:10.1371/journal.pntd.0008012.
Rios, T., Bomfim, L., and Ramos, I. (2021). The transition from vitellogenesis to choriogenesis triggers the downregulation of the UPR sensors IRE1 and PERK and alterations in the ER architecture in the follicle cells of the vector Rhodnius prolixus. *Cell Tissue Res.* doi:10.1007/s00441-021-03547-z.
Schmidt-Nielsen, K. (1997). *Animal physiology: adaptation and environment*. Cambridge university press.
Silva-Oliveira, G., De Paula, I. F., Medina, J. M., Alves-Bezerra, M., and Gondim, K. C. (2021). Insulin receptor deficiency reduces lipid synthesis and reproductive function in the insect Rhodnius prolixus. *Biochim. Biophys. Acta (BBA)-Molecular Cell Biol. Lipids* 1866, 158851.
Sterkel, M., Perdomo, H. D., Guizzo, M. G., Barletta, A. B. F., Nunes, R. D., Dias, F. A., et al. (2016). Tyrosine detoxification is an essential trait in the life history of blood-feeding arthropods. *Curr. Biol.* 26, 2188–2193.
Vieira, P. H., Benjamim, C. F., Atella, G. C., and Ramos, I. (2021). VPS38/UVRAG and ATG14, the variant regulatory subunits of the ATG6/Beclin1-PI3K complexes, are crucial for the biogenesis of the yolk organelles and are transcriptionally regulated in the oocytes of the vector Rhodnius prolixus. *PLoS Negl. Trop. Dis.* 1, 1.
Vieira, P. H., Bomfim, L., Atella, G. C., Masuda, H., and Ramos, I. (2018a). Silencing of RpATG6 impaired the yolk accumulation and the biogenesis of the yolk organelles in the insect vector R. prolixus. *PLoS Negl. Trop. Dis.* 12, e0006507. doi:10.1371/journal.pntd.0006507.
Vieira, P. H., Bomfim, L., Atella, G. C., Masuda, H., and Ramos, I. (2018b). Silencing of RpATG6 impaired the yolk accumulation and the biogenesis of the yolk organelles in the insect vector R. prolixus. *PLoS Negl. Trop. Dis.* 12. doi:10.1371/journal.pntd.0006507.
Walter-Nuno, A. B., Oliveira, M. P., Oliveira, M. F., Gonçalves, R. L., Ramos, I. B., Koerich, L. B., et al. (2013). Silencing of maternal heme-binding protein causes embryonic mitochondrial dysfunction and impairs embryogenesis in the blood sucking insect Rhodnius prolixus. *J. Biol. Chem.* 288, 29323–29332.
World Health Organization (2019). WHO. Available at: https://www.who.int/en/news-room/fact-sheets/detail/chagas-disease-(american-trypanosomiasis).
Zhang, D.-W., Xiao, Z.-J., Zeng, B.-P., Li, K., and Tang, Y.-L. (2019). Insect behavior and physiological adaptation mechanisms under starvation stress. *Front. Physiol.* 10, 163.
